# A new versatile x–y–z electrospinning equipment for nanofiber synthesis in both far and near field

**DOI:** 10.1038/s41598-022-08310-0

**Published:** 2022-03-22

**Authors:** Mar Calzado-Delgado, M. Olga Guerrero-Pérez, King Lun Yeung

**Affiliations:** 1 Department of Chemical and Biological Engineering, The Hong Kong University of Science and Technology, Clear Water Bay, Kowloon, Hong Kong SAR; 2 Department of Chemical Engineering, Universidad de Málaga, E29071 Malaga, Spain; 3 Division of Environment and Sustainability, The Hong Kong University of Science and Technology, Clear Water Bay, Kowloon, Hong Kong SAR; 4 HKUST Shenzhen-Hong Kong Collaborative Innovation Research Institute, Futian, Shenzhen, Guangdong China

**Keywords:** Engineering, Materials science, Nanoscience and technology

## Abstract

This work describes a versatile electrospinning equipment with rapid, independent, and precise x–y–z movements for large-area depositions of electrospun fibers, direct writing or assembly of fibers into sub-millimeter and micron-sized patterns, and printing of 3D micro- and nanostructures. Its versatility is demonstrated thought the preparation of multilayered functional nanofibers for wound healing, nanofiber mesh for particle filtration, high-aspect ratio printed lines, and freestanding aligned nanofibers.

## Introduction

Nanomaterials, particularly nanofibers, are the subject of intense studies because of their potential uses in medicine, electronics, and catalysis^1–3^. Indeed, nanofibers serve as architectural elements in cell and tissue scaffolds^4–6^ and are a basis for artificial skins and intelligent textiles^7,8^. In addition, they are also an efficient delivery system for bioactive molecules in therapeutic applications. This growing interest drives research into their fabrications through self-assembly, phase-separation drawing process, template-assisted synthesis, solvent casting methods, and electrospinning^9–11^. Notably, electrospinning produces micro and nanofibers of various materials, morphologies, textures, and porosities^12,13^. Its versatility allows natural, synthetic, and composite nanofibers to be electrospun and produced in scalable quantities. Moreover, the assembly of the nanofibers can be guided to create complex 3D objects for various application needs^14,15^. Thus, electrospun nanofibers are used in drug delivery, wound dressing, bone regeneration, and tissue engineering research^16^.

There are numerous improvements in the design of electrospinning equipment to enhance its performance. Investigating the whipping instability of the Taylor cone during electrospinning, Xu et al.^17^ propose a rigorous mathematical approach in predicting jet behavior and the use of a magnetic field to control the electrospinning process. Alternatively, Rebrov et al.^18^ employed high-voltage pulse generator to deposit aligned fibers, while Coppola et al.^19^ described a tethered pyro-electrospinning technique for direct printing electrospun fibers. Furthermore, You et al.^20^ used a positively-charged, sharp-pin electrode to modify and redistribute electric charge on the base plate to control the jet trajectory and nanofiber deposition. Finally, Thomas et al.^21^ use a 3D-printing platform to demonstrate tri-axial controlled electrospinning. Coaxial, emulsion, and reactive electrospinning processes directly manipulate the fiber morphology and chemistry and extent their applications to natural and inorganic materials^22,23^.

A new electrospinning device was designed to seamlessly transition from large-area, far-field electrospinning to high-precision, near-field fiber printing. It is built to craft and assemble nanofibers into complex objects such as meshes, nets, nano-walls, aligned fibers, and multi-layered, thin films from a variety of materials, including hydroxypropyl methylcellulose (HPMC), polyethylene oxides (PEO), and polyvinyl alcohol (PVA) materials that are commonly used in food, drug, and biomaterial research and applications.

## Results and discussion

### Design and set-up of the electrospinning equipment

**Figure 1:**
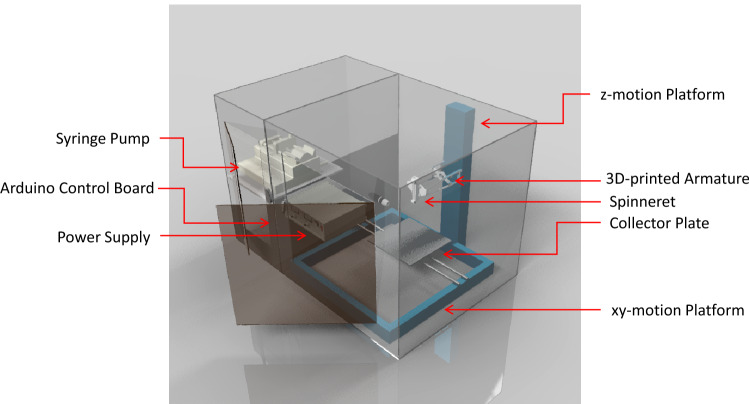
An illustration of the versatile electrospinning equipment for large-area, high precision deposition, writing, and printing of electrospun nanofibers.

A drawing of the electrospinning equipment is presented in Fig. 1. The equipment is designed for rapid and independent x-y-z movements without compromising accurate positioning and precision movements to allow its versatile use for depositing electrospun fibers over large area, writing and assembling fibers into precise sub-millimeter patterns with micron-size features, or precision printing of 3D microstructures. Besides precision movements, an accurate control over the electric field is a must to not only control fiber deposition but also its diameter and size. This combination is responsible for highly reproducible fiber production.

### Large-area electrospinning

**Figure 2:**
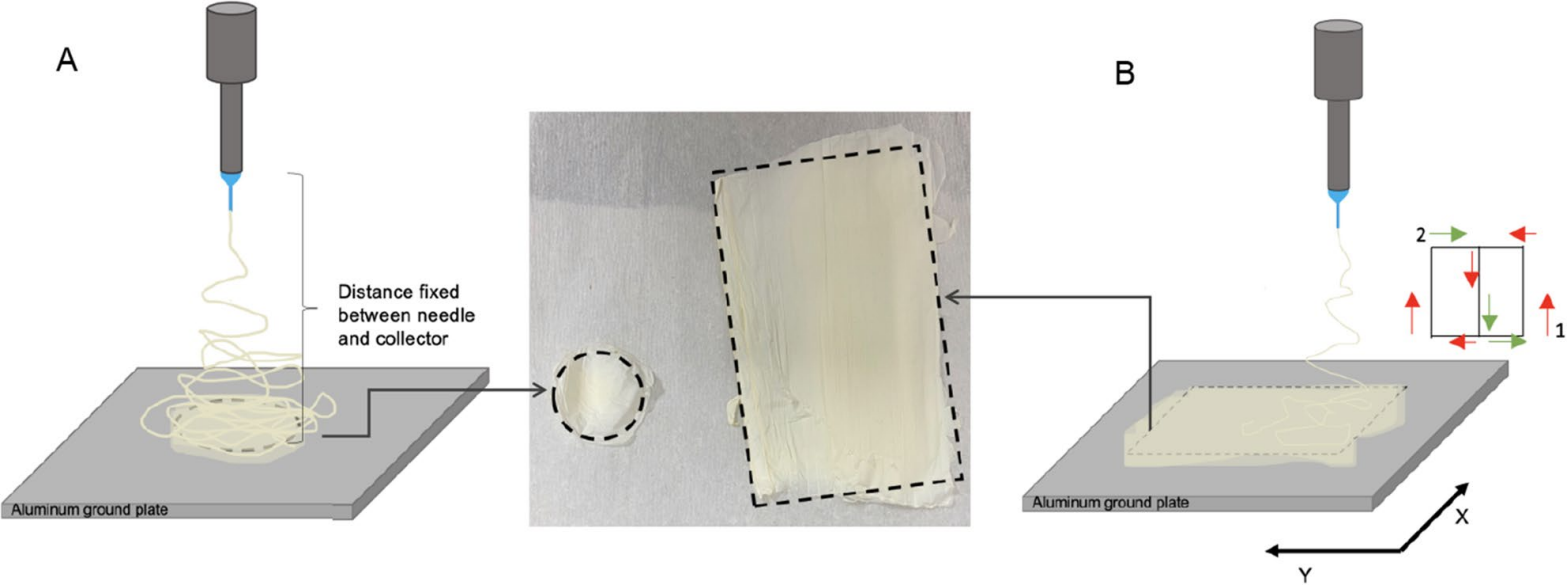
Large-area deposition of electrospun fibers under different x–y collector base movements, (**A**) fixed or rotational motions and (**B**) raster motion. Note: 10% HPMC in DDI water at 0.1 mL/h flow; 7.3 kV, and spinneret height (z) of 11 cm.

**Figure 3:**
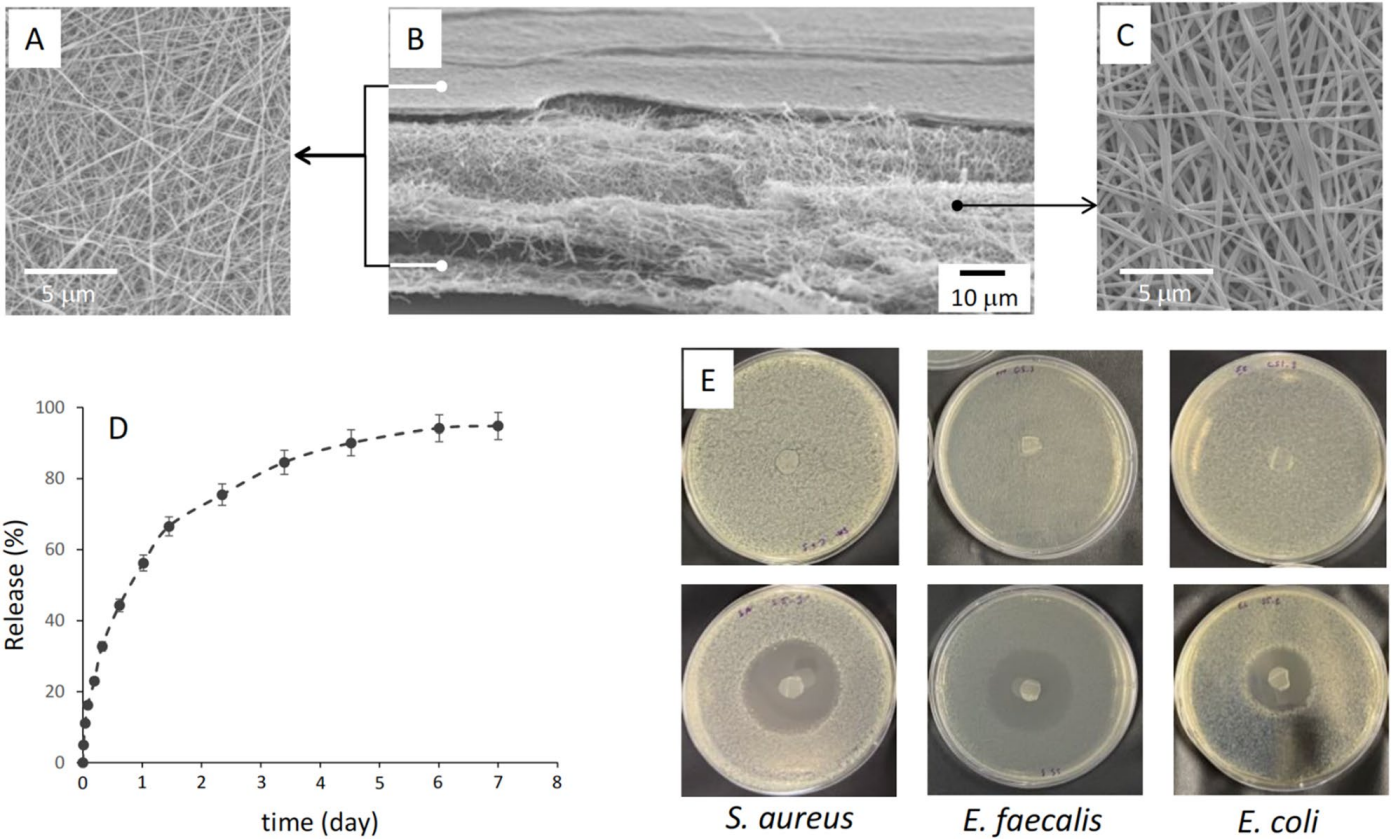
Scanning electron micrographs of HPMC/PVA/HPMC with PVA nanofibers loaded with 10 wt.% amoxicillin/clavulanic acid with (**A**) top HPMC layer, (**B**) sample cross-section, and (**C**) PVA sandwiched layer. (**D**) Amoxicillin/clavulanic acid release plot over 7 days and (**E**) Kirby-Bauer disc diffusion assay of HPMC/PVA/HPMC without (control, top) and with 10 wt.% amoxicillin/clavulanic acid (treatment, bottom) for *S. aureus, E. faecalis,* and *E. coli*.

Electrospun fibers can be deposited over a large area to form a fiber mat, as shown in Fig. 2. The x-y movement of the collector base can be programmed to obtain different sample sizes and shapes. For example, a rotational plate motion in Fig. 2A defines a circular deposition area as shown in Fig. 2A inset. Similarly, programming a raster movement (Fig. 2B) deposits a rectangular fiber mat on the collector base, as shown in Fig. 2B inset. It ensured uniform deposition and thickness across the entire electrospun sample. Thus, it is possible to seamlessly electrospun multiple layers of functional fibers, as illustrated in Fig. 3.

Figure 3B present an electrospun PVA (polyvinyl alcohol) fiber loaded with 10 wt.% amoxicillin and clavulanic acid (Fig. 3C) sandwiched between HPMC (hydroxypropyl methylcellulose) layers (Fig. 3A). The HPMC nanofibers are hypoallergenic and have excellent biocompatibility and skin adhesion ^24,25^, while PVA is an excellent matrix for storing and releasing drug molecules^26,27^. The two HPMC layers are 5.2 μm thick and consist of 110 ± 20 nm nanofibers to provide structural support for the 30.5 mm thick electrospun PVA (200 ± 40) nm. The PVA nanofibers have an area-to-volume ratio of 2 x 10^7^ m^-1^ and sustain a gradual release of amoxicillin/clavulanic acid with over 94.8% of the stored active components released at the end of the seven days, as shown by Fig. 3D. In addition, it endowed the material with excellent bactericidal activities against Gram-positive (i.e., *S. aureus* and *E. faecalis*) and Gram-negative bacteria (i.e., *E. coli*), as shown in Fig. 3E. 3-(4,5-dimethylthiazol-2-yl)-2,5-diphenyl-2H-tetrazolium bromide (MTT) cell activity and morphological identity assays on A431 human epidermoid carcinoma epithelial cells indicate excellent biocompatibility and cell viability on the HPMC/PVA/HPMC multilayer sample. Used as a wound dressing, it prevents infection and promotes wound healing, as observed on 5 weeks old female C57BL/6J mice (Table 1).

**Table 1:** Wound healing properties.

Samples^1^	Wound size (cm^2^)				Wound healing (%)	
	Day 0	Day 3	Day 7	Day 14	Day 7	Day 14
Control	0.70 ± 0.1	0.64 ± 0.1	0.33 ± 0.1	0.25 ± 0.1	47 ± 0.1	65 ± 0.1
Treatment	0.63 ± 0.2	0.62 ± 0.1	0.12 ± 0.1	0.005 ± 0.1	19 ± 0.1	99 ± 0.1

^1^PVA nanofiber serve as control and HPMC/PVA/HPMC with 10 wt.% amoxicillin and clavulanic acid (Fig. 3) is used for treatment arm of the study.

**Figure 4:**
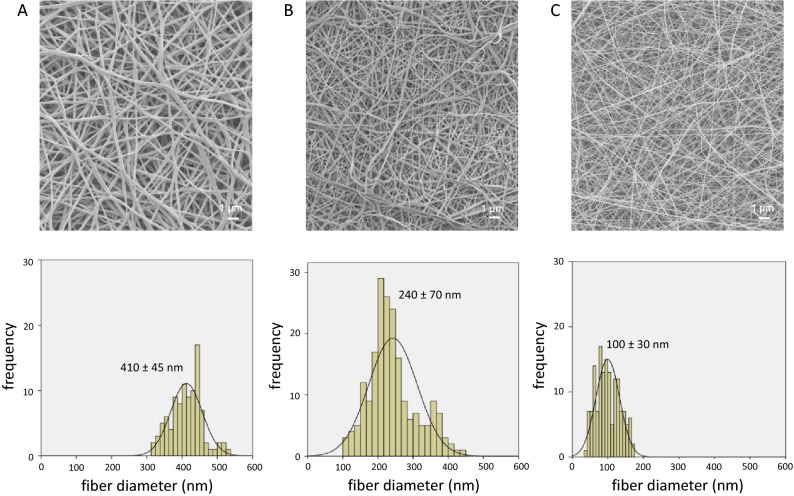
Scanning electron micrographs and fiber diameter distribution for HPMC nanofibers electrospun from 1.5 wt.% HPMC dissolved in H_2_O:EtOH solvent with mass ratio of (**A**) 2.0, (**B**) 1.5, and (**C**) 1.0.

The HPMC nanofibers of different diameters can be electrospun by varying the solvent mass ratio of H_2_O: EtOH from 2.0 to 1.5 and 1.0 to give 400, 250, 100 nm fibers as shown in Fig. 4 and Supporting Information. HPMC has good solubility in an appropriate mixture of water and ethanol. Moreover, ethanol has a lower surface tension, faster evaporation rate, and is slightly (ca. 20%) more viscous than water. It, therefore, favors the preparation of thinner nanofibers. Indeed, adding more ethanol (i.e., H_2_O: EtOH from 2.0 to 1.5) sharply decreased the fiber diameter by roughly 150 nm from 410 ± 45 nm (Fig. 4A) to 240 ± 70 nm (Fig. 4B) and mean pore diameters of 210 ± 110 nm and 170 ± 60 nm, respectively. An HPMC dissolved in 1 H_2_O:1 EtOH solution produces thin nanofibers (Fig. 4C) with a mean diameter of 100 ± 30 nm and a mean pore diameter of 140 ± 50 nm. It has the smallest pores among the three samples and the best drug dosing characteristics. Therefore, it serves as the HPMC layers in the HPMC/PVA/HPMC multilayer sample presented in Fig. 3.

### Near-field nanofiber writing

**Figure 5:**
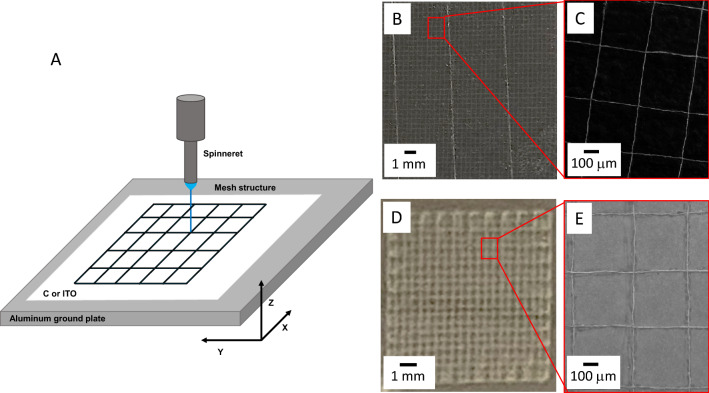
(**A**) Schematic drawing of the near-field writing and assembly of single strand nanofiber into sub-millimeter mesh pattern on conducting (**B**) and (**C**) carbon and (**D**) and (**E**) ITO glass. (**B**) Optical microscope picture of 20 × 20 mm^2^ mesh with (**C**) scanning electron microscope (SEM) image showing 120 nm PEO nanofiber with a line-to-line grip pitch of 500 mm. Note: The near-field writing was done at a 2 kV and 3 mm spinneret height (z) at a writing speed of 18 mm/s. (**D**) Optical microscope image of a 10 × 10 mm^2^ mesh and (**E**) scanning electron micrograph (SEM) showing 150 nm PEO nanofiber with a line-to-line grip pitch of 500 mm. *Note* The near-field writing was done at a 3.2 kV and 4 mm spinneret height (z) at a writing speed of 15 mm/s. The z-axis varies ± 10 mm to maintain electrical field.

The versatility of the new equipment is demonstrated in Fig. 5, where a single strand of 120 nm PEO nanofiber was precisely drawn into a 500 mm mesh (Fig. 5A) covering a 20 mm × 20 mm area of the carbon substrate as shown by the optical microscope picture in Fig. 5B. A closer inspection shows the strands are perfectly aligned and intercepting perpendicularly (i.e., 90 ± 2°) at the nodes to form a square opening, as shown in Fig. 5C. Individual strand does appear to twist a little bit, resulting in slight slack. However, the sides of the mesh opening deviate by less than 10% from 500 mm. The entire pattern is written in 100 s at a speed of 18 mm/s. Similarly, PEO nanofibers can be directly written on conducting ITO glass (Fig. 5D), demonstrating its potential in microelectronic fabrication. Here, thicker PEO strands were electrospun to create a similar mesh pattern over a 10 mm × 10 mm area. Finally, these 150 nm PEO nanofibers were laid down in a regular grid pattern, as shown in Fig. 5E. A strong electric field and fast writing speed give nanofibers of narrow diameter. It is done by increasing applied voltage, lowering spinneret height, and increasing x–y movements.

**Figure 6:**
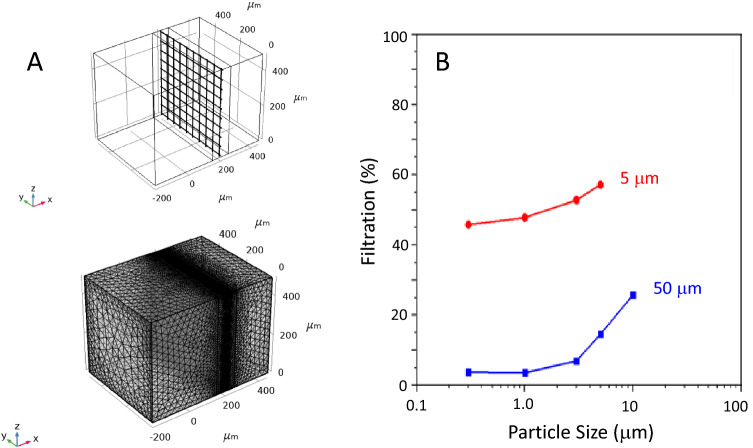
(**A**) 50-mm square mesh geometry and computational mesh size and (**B**) calculated filtration efficiency from the computational simulation for 50 and 5 mm mesh for different airborne particle sizes. *Note* Fiber and particle Poisson ratio of 0.29 and 0.32, fiber and particle Young’s moduli of 3.2 × 10^9^ Pa and 2.0 × 10^11^ Pa, face velocity is 0.105 m/s, air dynamic viscosity and density of 0.9 × 10^–5^ Pa $\cdot s$ and 1.225 kg/m^3^.

It is possible to detach and lift the electrospun mesh from the substrate as a sieve filter for particles. It would represent an ideal capture surface for investigating particulate filtration, especially of aerosols and droplets with sizes of 0.1 to 10 μm responsible for the airborne transmission of respiratory diseases. The computational calculations in Fig. 6 show that filtration efficiency will depend on the mesh opening size. The simulation done in Comsol Multiphysics was conducted for mesh openings of 50 and 5 microns, and for particle sizes ranging from 0.3 to 10 microns. The filtration efficiency increases with finer mesh openings and larger particulates. The results demonstrate that even coarse filter mesh openings of 5 μm (Fig. 6A) can reach MERV10 standards at higher flow and lower pressure drops (Fig. 6B). Particulate capture also relies on nanofiber’s material and surface properties as it is governed by the molecular interactions between particles and surfaces, and the capture rate can be enhanced by increasing particle adhesion and minimizing rebound and resuspension.

### Near-field printing of 3D micro- and nanostructures

**Figure 7:**
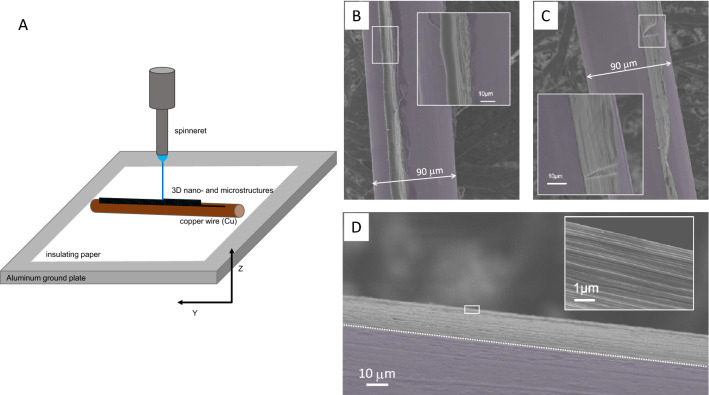
(**A**) Schematic drawing of the near-field printing of (**B**) PEO micro- and (**C**) and (**D**) nanostructures on conducting copper wire. Scanning electron micrographs of (**B**) a PEO line feature with height-to-width aspect ratio of 5 by repeated printing (8.6 ± 0.08) µm electrospun PEO atop one another at 2.6 kV, 4 mm spinneret height with programmed z-axis upward movement of 10 µm every 10 s, and printing speed of 18 mm/s, (**C**) a 50 height-to-width aspect ratio PEO line from 50 aligned strands of (440 ± 16) nm PEO nanofibers printed by varying the voltage from 2.0 to 3.4 kV at a spinneret height of 3 mm with an upward z-movement of 10 µm every 15 s, and printing speed of 24 mm/s, and (**D**) a ultra-high aspect ratio (142) PEO line feature obtained by aligned printing of 140 strands of (102 ± 10) nm PEO nanofibers by varying the voltage from 1.5 to 3.1 kV, 2 mm spinneret height with programmed upward z-movement of 10 µm after each 20 s, and printing speed of 30 mm/s.

Figure 7 displays examples of near-field printing of PEO nanofiber on a copper wire surface. The 90 µm diameter wire is 8 cm long and held in place as illustrated in Fig. 7A. Along the length of the wire, strands of PEO nanofibers were printed atop each other to create a narrow wall. The spinneret height (z-axis) was positioned 4 mm above the wire for printing the five strands of 8.6 ± 0.08 µm PEO fiber in Fig. 7B. The spinneret was programmed to move a 10-mm step upward every 10 s while the voltage was varied from 3.0 to 3.6 kV during printing to produce the 43 ± 0.1 µm high printed feature (Fig. 7B inset). The printing was done at a speed of 18 mm/s. A slight warping observed under the scanning electron microscope was an artifact caused by sample preparation. Fifty layers of 440 ± 16 nm PEO nanofibers shown in Fig. 7C were printed at a speed of 23 mm/s and a starting spinneret height of 3 mm with incremental upward z-movement of 10 µm per 15 s. The voltage was varied from 2.0 to 3.4 kV during printing. An incision made on the printed sample shows perfect alignment of the nanofibers along the entire thickness (Fig. 7C inset). Figure 7D shows a printed nanostructure consisting of 140 printed strands of 102 ± 10 nm PEO nanofiber on the copper wire. It was printed at a high speed of 300 mm/s with the z-axis adjusted upward by 10 µm every 20 s. Despite its narrow width, the thin nanofiber filaments are perfectly aligned, as shown in Fig. 7D inset. The freestanding feature is 102 nm thick and 14.3 ± 0.002 µm high giving it an aspect ratio of 142, while samples in Fig. 7B and C have aspect ratios of 5 and 50, respectively. This clearly demonstrates the new electrospinning equipment can print 3D micro- and nanostructures with control and precision.

**Figure 8:**
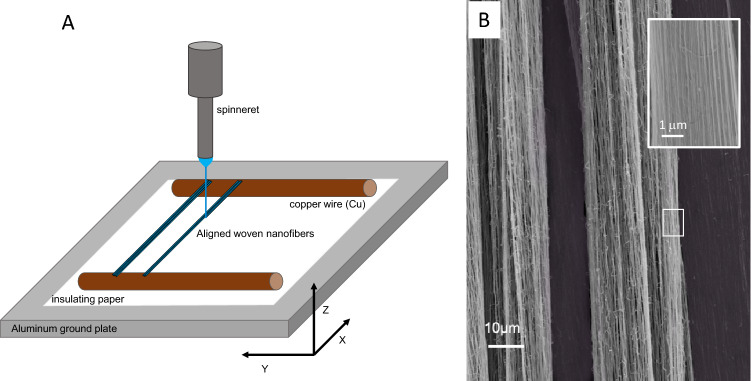
(**A**) Schematic drawing of the near-field electrospinning of PVA nanofiber between parallel copper wires to create (**B**) aligned strands 130 ± 12 nm PVA nanofiber by varying the voltage between 1.8 and 2.8 kV, spinneret height of 3 mm, x-axis movement speed of 20 mm/s and y-axis movement of 20 µm/min.

Figure 8 illustrate near-field electrospinning could be used to weave freestanding nanofibers between two copper wires acting as warp beams in a miniature “loom”. Here, PVA nanofibers were strung between the wires at a rapid speed of 20 mm/s along the x-axis with the y-axis being offset to the left at 20 µm per minute to create the suspended aligned nanofibers in Fig. 8B. The scanning electron micrograph clearly shows each individual strand is separate and has a diameter of 130 ± 12 nm. It is robust and can be handled and prepared for microscopy examination. It is possible to program the near-field electrospinning process to weave more complex design to create functional textile mats from different electrospun nanofiber materials.

## Conclusions

The versatile electrospinning equipment can seamlessly operate in both far-field and near-field conditions, made possible by the rapid but precise x-y-z movements with accurate positioning and electrical field control that enables large-area deposition of electrospun fibers, direct writing of nanofibers into sub-millimeter and micron-size patterns, and printing of 3D micro- and nanostructures. The equipment was successfully used to prepare multilayered functional nanofibers for biomedical application of wound healing. In addition, submillimeter square mesh made of nanofibers were printed over an extended area for studying dynamics of particulate capture and filtration. Finally, high aspect ratio nanofiber lines with narrow width were printed at high speed to demonstrate 3D nano-printing process.

## Methods

### Electrospinning equipment

The base collector was mounted on a motion platform with independently *x* and *y* movements controlled by two sets of timing belt, screw shaft, and stepper motor (SKU:81042, 42BYG) through a main board connected to Arduino (Megablock MegaPi) and a PC computer that enable rapid long-distance movements (100 mm/s), precise positioning (± 5 μm), and fine micron-step motions (1.8 ± 0.09°/PHASE and 400 steps/mm) for a seamless large-scale precision electrospinning. The movement can be programmed to deposit, write, or print different patterns of electrospun fibers. The z-movement controlled the distance of the spinneret from the collector and used a set of stepper motor and shaft to give precision movements as small as 5 mm. The interchangeable spinneret assembly was supported by a cantilevered 3D printed arm that also held a light source and a USB camera (LED Torch Lighting EVER, 8 LED USB camera microscope x1000 magnification) for monitoring the electrospinning. The base and the spinneret were connected to a programmable power supply (NanoNC PS355) and calibrated to deliver precise electric field for controlled electrospinning. The spinneret was designed and fabricated in the laboratory and connected to a syringe pump (kdScientific KDS200) that delivers 0.004µL/h to 3520mL/h of electrospinning solutions. The electrospinning equipment was housed in a transparent chamber equipped with a set of DC fan and humidifier/dehumidifier units to maintain the temperature and humidity during electrospinning. The chamber is connected to a local exhaust to vent solvents. Detailed description of the electrospinning equipment and operation is available in Supplementary Information. 

### Materials and preparations

Hydroxypropyl methylcellulose (HPMC, MW = 120,000 grams per mole), polyvinyl alcohol (PVA, MW = 1,250,000 grams per mole), and poly(ethylene oxide) (PEO, MW = 300,000 grams per mole) were acquired from Sigma-Aldrich and were used as received. Ethanol (Absolute, Analar Normapur) and distilled deionized (DDI) water were used as solvent. Table 2 lists the composition of the electrospinning solution of HPMC, PEO, and PVA used to produce the samples shown in Figs. 3, 4, 5, 6. The electrospun fiber mat was collected on glossy paper, written fiber patterns on conducting carbon and ITO glass, and the printed fibers use thin copper wires (Ø 95 µm and 8 cm length) as printing guide. Electrospinning operations were conducted at room temperature (ca. 20 °C) and 65% relative humidity.

**Table 2:** Compositions of electrospinning solutions.

Polymer	Polymer (wt.%)	Solvent		Spinneret flow (ml/h)	Voltage (kV)	Distance (cm)	Fiber diameter (nm)
		H_2_O	EtOH				
HPMC (cf. Fig. 3A)	1.5	0.50	0.50	0.50	12	15.00	110 ± 40
PVA (cf. Fig. 3C)	7	1.0	0.0	0.10	7.2	13.00	220 ± 30
HPMC (cf. Fig. 4A)	1.5	0.67	0.33	1.50	7.0	15.00	410 ± 45
HPMC (cf. Fig. 4B)	1.5	0.60	0.40	0.80	5.8	11.00	240 ± 70
HPMC (cf. Fig. 4C)	1.5	0.50	0.50	0.18	9.3	13.00	100 ± 30
PEO (cf. Fig. 5)	10	1.0	0.0	0.008	3.2	0.30	118 ± 10 (carbon) 146 ± 15 (ITO)
PEO (cf. Fig. 7B)	10	1.0	0.0	0.008	2.6	0.40	8600 ± 80 (Cu wire)
PEO (cf. Fig. 7C)	10	1.0	0.0	0.008	2.0	0.30	440 ± 16 (Cu wire)
PEO (cf. Fig. 7D)	10	1.0	0.0	0.008	1.5	0.20	102 ± 10 (Cu wire)
PVA (cf. Fig. 8)	10	1.0	0.0	0.010	1.8	0.30	130 ± 12

### Material characterization

The electrospun samples were inspected under an Olympus BH2-MJLT optical microscope prior to sample preparation for scanning electron microscopy. Samples from electrospun fiber mat were cut by sharp pair of scissors and mounted on aluminum specimen stub by a conducting carbon adhesive tape. A light sputter coating of gold in K575xd sputtering equipment prevents sample charging. The written and printed fibers were also mounted, and sputter coated with an additional precaution of using conductive adhesives to ground the sample surface. The samples were observed under JSM-6700F and JSM-7100F at 10 kV The images were analyzed, and the diameter of the nanofibers were measured by Image J and processed using IBM SPSS Statistics 23 software.

### Bactericidal and wound healing studies

The HPMC/PVA/HPMC with 10 wt.% amoxicillin and clavulanic acid was investigated for drug release, bactericidal, and wound healing. The release of active ingredient was measured on 30 mg sample in 30 mL PBS solution at 37 °C over 7 days. Aliquots were taken at fixed time intervals and analyzed by Agilent UV Vis spectrophotometer and the released Amoxicillin/Clavulanic Acid were quantified against standard calibration. MTT assay was used to measure the cell viability, proliferation, and cytotoxicity of the sample on A431 human epidermoid carcinoma epithelial cells. Kirby-Bauer disc diffusion determined the *S. aureus*, *E. faecalis*, and *E. coli* to the HPMC/PVA/HPMC with 10 wt.% amoxicillin and clavulanic acid and control PVA sample. The 1 cm diameter test samples were obtained by biopsy punch and placed in the middle of the Mueller- Hinton agar petri dish seeded with a 10^8^ CFU/ml bacterial suspension (0.5 McFarland Standard). The inhibition zones were measured after incubating at 37°C for 48 h. Five weeks old C57BL/6J mice were individually caged and fed with sterile rodent chow. A cocktail of ketamine/xylene (80-100mg/Kg and 10-12.5 mg/kg) was injected as anaesthesia and a sterile biopsy punch was used to create a round-shaped wound. The wound was dressed with PVA (control) or HPMC/PVA/HPMC with 10 wt.% amoxicillin and clavulanic acid (treatment). Painkiller (ketoprofen 100mg/mL) was added to the drinking water to relieve pain in animals the day after the procedure. The wound of each mouse was photographed and measured at day 0, 3, 7 and 14 of the procedure.

## Supplementary Information

Supplementary Information.
