# Supplementary material for: A new versatile x–y–z electrospinning equipment for nanofiber synthesis in both far and near field
Source: Sci Rep. 2022 Mar 22;12:4872. doi: 10.1038/s41598-022-08310-0 (PMC8940893; doi:10.1038/s41598-022-08310-0)
Supplement: Supplementary file 1 — Supplementary Information. [file 41598_2022_8310_MOESM1_ESM.docx]

**Supplementary Information**

A new versatile x-y-z electrospinning equipment for nanofiber synthesis in both far and near field

Mar Calzado Delgado1,2,+, M. Olga Guerrero-Pérez2, King Lun Yeung1,3,4,*.+

1 The Hong Kong University of Science and Technology, Department of Chemical and Biological Engineering, Clear Water Bay, Kowloon, Hong Kong SAR

2University of Málaga, Department of Chemical Engineering, Málaga, E-29071, Spain

3 The Hong Kong University of Science and Technology, Division of Environment and Sustainability, Clear Water Bay, Kowloon, Hong Kong SAR

4 HKUST Shenzhen-Hong Kong Collaborative Innovation Research Institute, Futian, Shenzhen, Guangdong, China

*[kekyeung@ust.hk](mailto:kekyeung@ust.hk)

+these authors contributed equally to this work


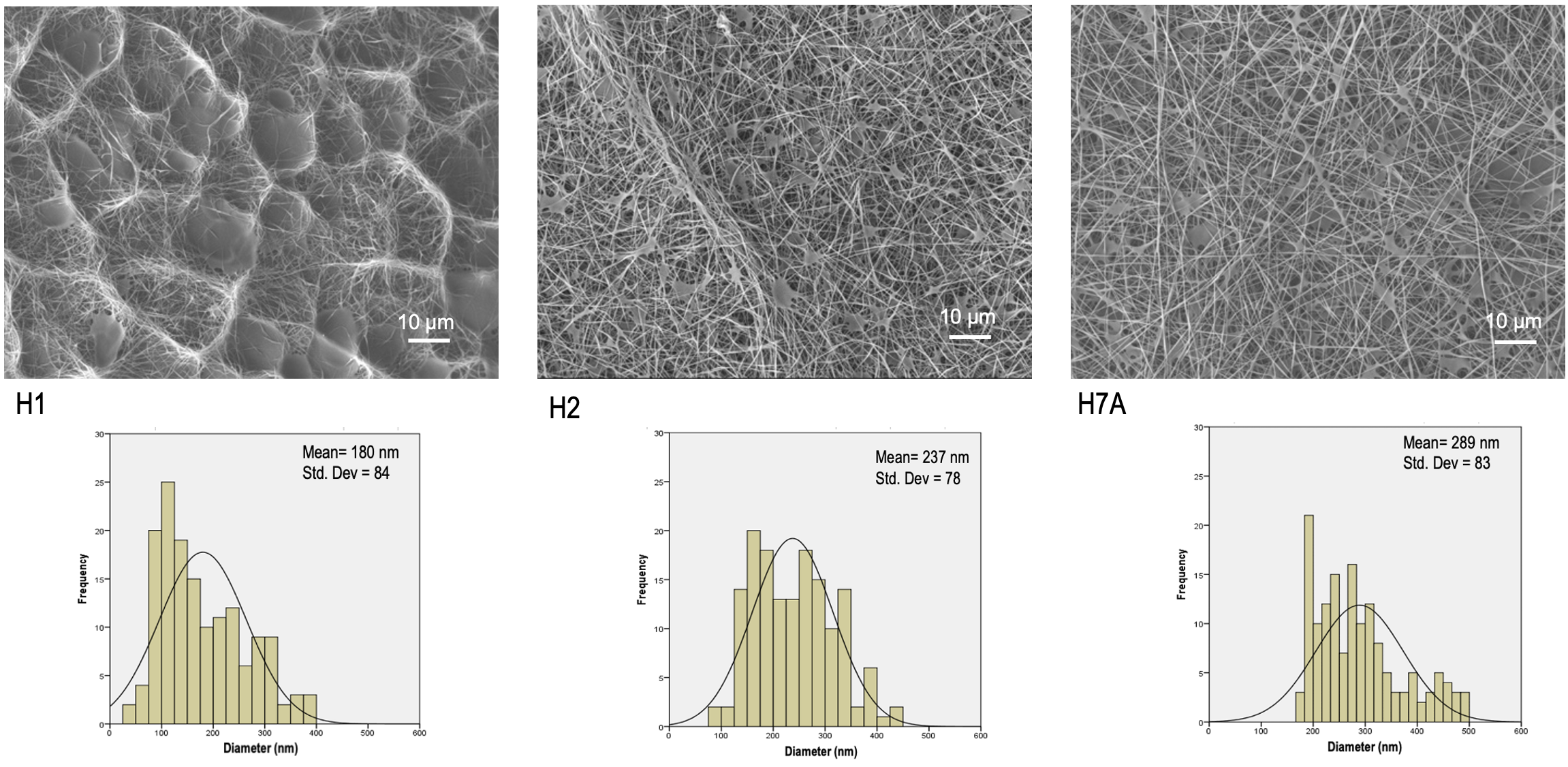


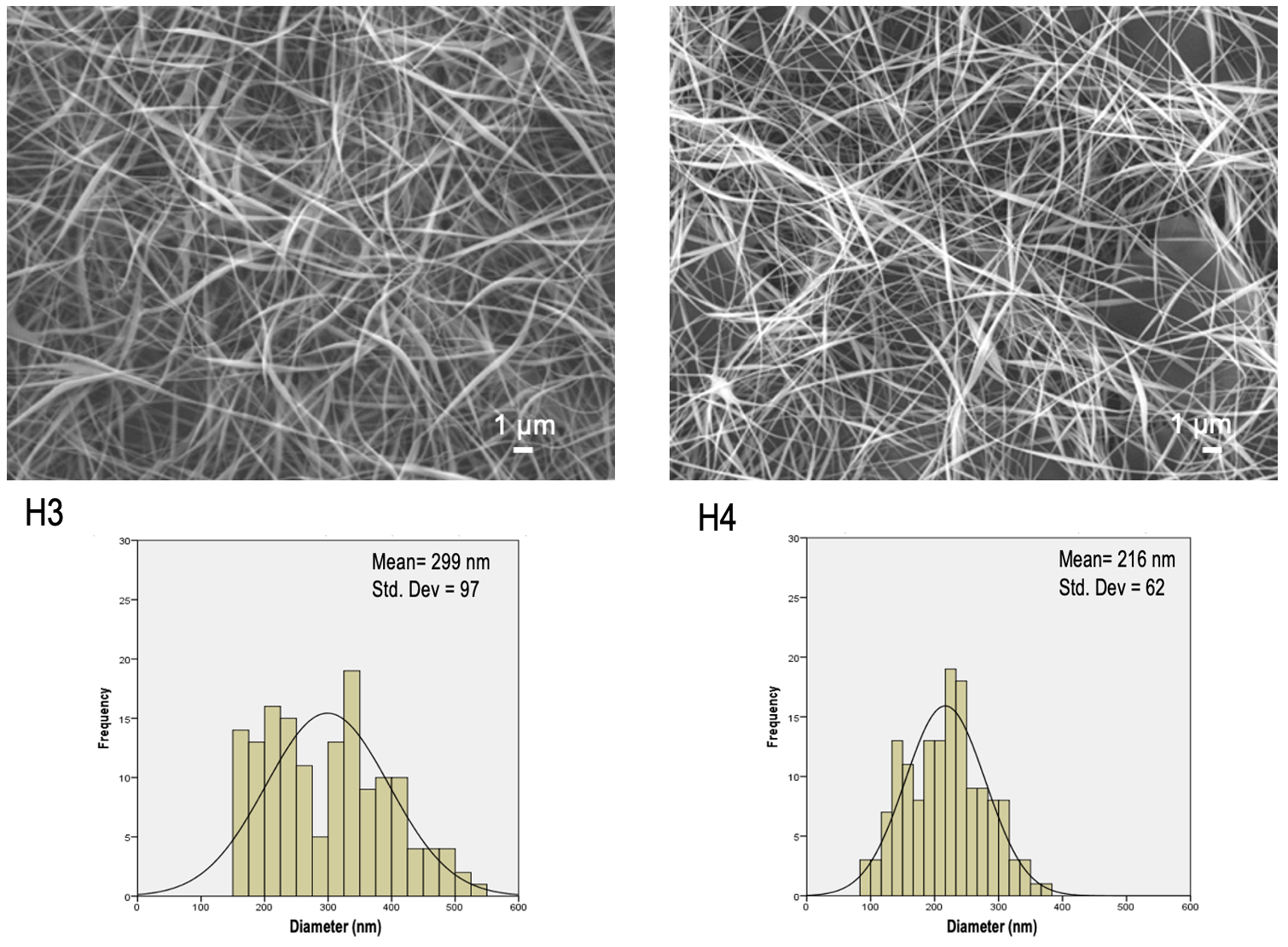

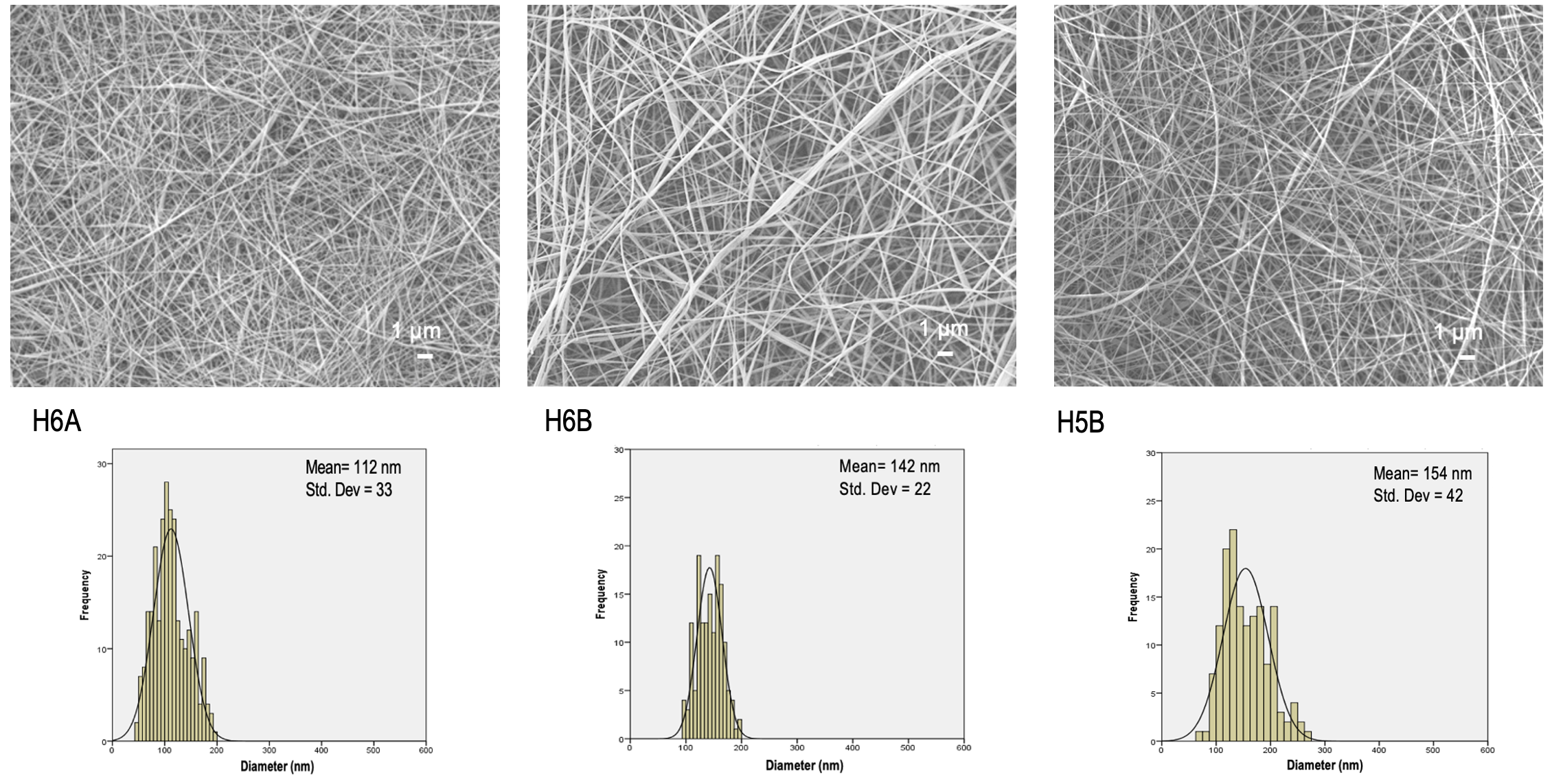


**Figure 1SI**: SEM images and diameter size distributions for H1, H2, H3, H4, H5B, H6A, H6B AND H7A samples. Description of synthesis and working parameters used described in table 1.

**Specifications of the Electrospinning equipment. Assembly components.**

| 4 x Beam 0824-496 | 6 x Linear Motion Shaft D8x496mm | 8 x Screw Countersunk M3x8 | 38 x Nut M4 |
| --- | --- | --- | --- |
| 2 x Beam 2424-504 | 1 x Linear Motion Shaft D4x512mm | 19 x Headless Set Screw M3x5 | 2 x Flange Bearing 4x8x3mm |
| 4 x Beam 0824-48 | 8 x Linear Motion Slide Unit 8mm | 7 x Shaft Collar 4mm | 6 x Timing Pulley 8T |
| 4 x Beam 0824-96 | 3 x Linear Motion Shaft D4x80mm | 8 x Flange Bearing 4x8x3mm | 5 x Bracket U1 |
| 4 x Beam 0824-112 | 7 x Plate 3x6 | 8 x Plastic Rivet 4060 | 2 x Bracket 3x3 |
| 2 x Beam 0824-80 | 24 x Screw M4x14 | 4 x Plastic Rivet 4100 | 1 x Plastic Ring 4x7x2 |
| 1 x Flexible coupling 4x4mm | 2 x Shaft Collar 4mm | 4 x Cuttable Linkage 3 | 6 x LS Bracket |
| 2 x D Shaft 4x56mm | 8 x Cross Recessed Pan Head Tapping Screws St2.2x9.5 | 6 x Micro Switch Buttom | 4 x Open-end Timing Belt (1.3m) |
| 30 x Screw M4x8 | 18 x Screw M4x30 | 3 x Stepper Motor Driver | 4 x Belt Connector |
| 21 x Screw M4x16 | 8 x Screw M4x22 | 3 x Me RJ25 Adapter | 1 x Base Board Plate |
|  | 3 x 42BYG Stepper Motor | 3 x 42BYG Stepper Motor Bracket V2.1 | 1 x MegaPi |

**Table 1SI:** Specifications and assembly components of the Electrospinning equipment

**Specifications and equipment resolution (42BYG Stepper Motor).**

Phase: 2PHASE

Step Angle: 1.8+-5%/PHASE

Rated Voltage: 12V

Current: 1.7A/PHASE

Resisrance: 1.5+-10%/PHASE

Inducrance: 2.8+-20%mH/PHASE

Holding Torque: 40N.cm Min

Detent Torque: 2.2N.cm Max

Insulation Class: B

Lead Style: AWG26 UL1007

Rotor Torque: 54G.cm2

Weight: 290.00 g

Steps per revolution: 200 steps (360/angle)

The resolution is 5 µm with values of tooth on the pulleys (8), step angle (1. 8º), stepping or stepper drivers’ microsteps (1/16th) and belt pitch (2mm).

These parameters can change in order to the working needs. The versality of the equipment allows to change or replace any components (electronic or metallic part) easily.

**Arm assembly of the equipment (Z-Axis) and ground plate as working area.**

Aluminium plate dimensions: 20 cm x 20 cm (the aluminium plate can be easily replaced for a bigger or smaller one).

Methacrylate plate as insulating plate, fixed with 2 cubic pieces of PLA on the mobile structure of the platform. The PLA pieces were printed using a Ultimaker 3 3D printer.


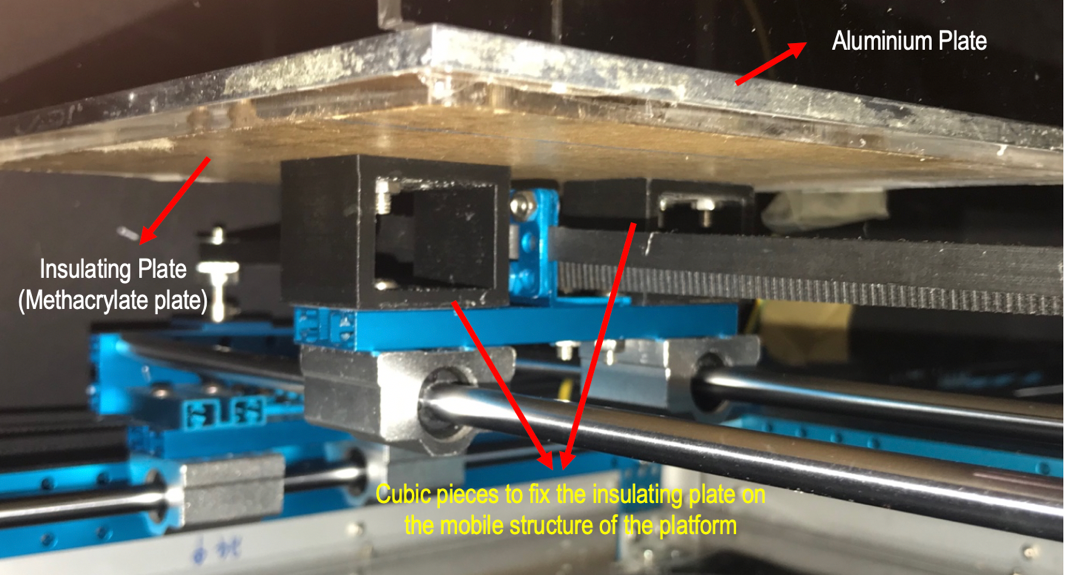


**Figure 2SI**: Assembly and components of the ground plate in the working area of the electrospinning equipment.


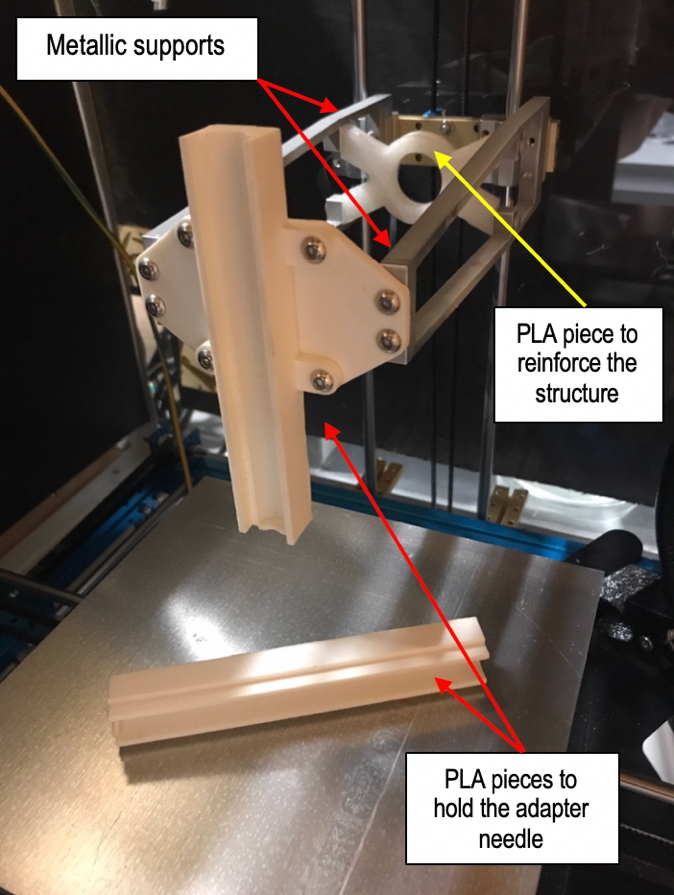


**Figure 3SI**: Arm assembly of the equipment which control Z-axis movement.
